# Different Behavior-Change Messaging Techniques Do Not Increase Customers’ Hand Sanitization Adherence During the COVID-19 Pandemic: A Natural Behavioral Study

**DOI:** 10.3389/fpsyg.2022.876131

**Published:** 2022-06-09

**Authors:** Lauren A. Booker, Emma L. Cordon, Hanne Sæderup Pedersen, Christina Fogtmann Fosgerau, Simon Egerton, Carina K. Y. Chan, Timothy C. Skinner

**Affiliations:** ^1^La Trobe Rural Health School, La Trobe University, Melbourne, VIC, Australia; ^2^Department of Nordic Studies and Linguistics, University of Copenhagen, Copenhagen, Denmark; ^3^Department of Computer Science and Information Technology, La Trobe University, Melbourne, VIC, Australia; ^4^School of Psychology and Public Health, La Trobe University, Melbourne, VIC, Australia; ^5^Department of Psychology, Centre for Health, and Society, University of Copenhagen, Copenhagen, Denmark

**Keywords:** Australia, COVID, public health, hand sanitization, coronavirus, pandemic, infection preventive behavior

## Abstract

**Introduction:**

Hand hygiene is an integral public health strategy in reducing the transmission of COVID-19, yet the past research has shown hand hygiene practices among the public is sub-optimal. This study aimed to (1) quantify hand sanitization rates among the public to minimize the transmission of COVID-19 and (2) evaluate whether different public health messaging, based on various behavior-change theories influences hand hygiene behavior in a natural setting.

**Methods:**

An observational, naturalistic study design was used with real-time customer activity data recorded against hand sanitizer usage in a regional hardware store. Primary outcome from the study was to measure the usage ratio by counting the amount of activity versus usage of hand sanitizer per hour against individual messages based on their behavioral change technique (BCT).

**Results:**

There was no significant difference between the baseline message and any of the intervention messages [*F*(16,904) = 1.19, *p* = 0.279] or between BCT groups [*F*(3,906) = 1.33, *p* = 0.263]. *Post hoc* tests showed no significant difference between messages (social comparison, *p* = 0.395; information, *p* = 1.00; and action planning, *p* = 1.00).

**Conclusion:**

This study showed that even during a pandemic, hand hygiene usage rates in a public setting were similar to the past studies and that compliance did not shift dependent on the public message displayed. This raises questions on whether requirements imposed on businesses to provide hand sanitizer to patrons are an ineffective and maybe an unnecessary economic burden. A measured approach to risk and behavioral analysis surrounding the use of hand sanitizer in a pandemic is suggested as a better approach to inform public policy on the value of hand sanitizer.

## Introduction

Hand hygiene has a direct, cost-effective impact on reducing the transmission of various diseases and infections (Borghi et al., 2002). The need for improved hand hygiene has been brought into focus by the COVID-19 global pandemic and is regarded as the single most important public health strategy in limiting its spread [Centers for Disease Control and Prevention (CDC), 2020; World Health Organization, 2020]. The consequences of poor hand hygiene practices during a pandemic are challenging, yet prior studies report consistently sub-optimal levels of routine hand hygiene behavior in public settings (Murray et al., 2009; Judah et al., 2010). For example, a recent pre-COVID-19 study reported 6.4% of hospital patrons used the hand dispensers on entering the building (Gaube et al., 2020), while an observational study conducted during the H1N1 influenza pandemic 2009, suggested that <20% of people entering a public hospital used hand sanitizer (Murray et al., 2009). If hand hygiene behavior is historically low among the public, then it is questionable how hand hygiene behavior may be increased during the COVID-19 pandemic. Since health messages and directives often accompany hand sanitizing dispensers, their content may influence people to increase hand sanitization in public settings, with the potential to reduce the transmission of COVID-19.

Health promotion is recognized for its importance in relation to public health strategies. In Australia, government-issued COVID-19 guidelines included the mandatory requirement for hand sanitizer dispensers in public settings (e.g., work settings, businesses, hospital, and retail). Yet, no explicit guidance was issued to increase the uptake of routine hand hygiene practice by the public. Previously, during both non-pandemic and pandemic contexts, behavior-change campaigns based on the psychological theories of behavior have positively influenced hand hygiene practices in a variety of public settings (Judah et al., 2009; Updegraff et al., 2011). In the context of measuring soap-use by patrons visiting a service-station restroom, Judah et al. (2009) found that health messages based on seven domains of behavior-change theory were associated with increases in hand hygiene behavior with notable gender differences. Furthermore, an observational study conducted during the 2009–2010 H1N1 pandemic by Updegraff and colleagues (2010; Updegraff et al., 2011) found gain-framed messaging increased hand sanitization usage rates by over 65%; however, these increases were measured from a relatively low usage baseline. Beyond the potential impact of behavior-change campaigns, there appears to be an over-reliance on the public’s ability to increase hand sanitizer usage during the heightened risk in a pandemic, and it is questioned whether this is enough. Since positive results have been reported on behavior-change messaging interventions in the past, investigating the most effective in optimizing hand hygiene behavior has important implications in reducing the spread of COVID-19 and other similar infectious diseases. Findings may be disseminated for easy uptake in the private and public sectors for increased hand sanitization rates.

Low rates of hand hygiene behavior among the public may increase the transmission of COVID-19 and impose unnecessary, economic burden on businesses required to provide hand sanitizer to patrons. Therefore, optimizing hand hygiene behavior in public settings, including workplaces, requires the understanding of current rates of hand sanitizer usage among the public and the further evaluation of sanitizer usage against health messaging based on various behavior-change theories. In the current COVID-19 pandemic context, the aim of this study, as the first known attempt in Australia, was to (1) measure customers’ non-enforced rate of hand sanitizer usage on entering a store during various levels of COVID-19 restrictions and (2) to determine whether public hand hygiene behavior may be increased using various behavior-change theories underpinning public health messaging. Particular attention will be given to how differences vary with the level of pandemic-driven restrictions, type of message, day of the week, and time of day.

## Materials and Methods

### Sample and Study Design

A natural behavior research design was used, with real-time customer activity data recorded against hand sanitizer usage to assess the impact of various BCT technique messages on hand hygiene behavior at a regional hardware store. Written consent was provided by the storeowners before the project commenced. Consent from customers was not needed as no identifying data was collected. The La Trobe University Human Research Ethics Committee (HREC) approved this low-risk application (HREC number HEC20265).

The Victorian State Government mandated that every business with on-site operations must have a “COVIDSafe” Plan, which included making soap and hand sanitizer available for all workers and customers throughout their site. Random spot checks were taking place across the state to ensure compliance. Failure to comply could result in on-the-spot fines of up to $9,913 or up to $20,000 for serious offenses (State Government of Victoria, 2021). In accordance with Australian Government COVID-19 restrictions, hardware stores were considered to be an essential service during the pandemic, so remained opened throughout the lockdown periods. The study was conducted in the regional city of Bendigo, Victoria, Australia, which experienced two state-based, hard lockdowns (Mar-Jun; Jul-Oct 2020) to combat the COVID-19 pandemic. During the data collection phase, cases in regional Victoria were low compared to Metropolitan areas, ranging from zero to 5–10 a day but the risk of infection was still high because at that time, no vaccinations were yet available. As such, the mandates and restrictions applied to the whole of the State. Bendigo is a regional city in the state of Victoria located approximately 150 km north-west of Melbourne, with a population of 153,092 (median age 42 years) and just under one fifth (17.6%) aged over 65 years. (Statistics ABo, 2016). The hardware store provided a natural setting in which to observe hand hygiene behavior and serviced the local population including families, professionals, technicians and trade workers, thus representing a wide spectrum of local demographics (Statistics ABo, 2016). The store also had established one main entrance and exit point, providing a naturalistic setting which allowed for nothing other than the introduction of the hand sanitizing stations in our study. A hand sanitizing dispenser was installed on a stand on the left-hand side, directly inside the store’s main entrance, with a 12-inch digital display monitor erected above the dispenser. Only the activity of customers entering the store was measured against their hand sanitizer usage.

### Measures

The hand sanitizer dispensers were customized with low power processing boards which increment an internal counter when sanitizer is dispensed. These boards communicated counts *via* the low power, long range network (LoRaWAN) and activity was reported within 16 s windows. The activity monitors were made of the same technology as the sanitizers and were equipped with a low power passive infrared sensor. The passive infrared element detects temperature differentials of four degrees when compared with ambient temperature. A trajectory across the sensors field of view that falls within this detection range was assumed to be a person crossing the sensors field of view. A record of this event is registered on an internal event counter, which in turn represents the number of people that have crossed the sensors field of view. Activity events are accumulated within 5-min windows and reported back *via* LoRaWAN. The display system used was low power e-ink display technology and used Zhuhai SUNY Technology 12-inch display screen. A raspberry PI 4 system equipped with a high precision real-time clock was used to run the display system and updated the displays hourly on the hour with a message selected randomly from a message database. If the random selection for the next display update matches the currently displayed message, then another message is randomly selected. All three systems were synchronized to the same real-time clock to allow for data alignment. Sanitizer data and activity monitor data were logged to secure cloud storage. The display system logged messages selected and was periodically collected manually *via* a USB stick connected to the raspberry PI system. The low power technology was used as all systems could then run independently from internal batteries over extended periods, in the order of months and years, and allows the flexible placement of sanitizers, activity monitors and displays.

### Procedure

At baseline, the message displayed on top of the dispenser simply stated a default descriptive message “hand sanitizer.” After the baseline period, a set of 14 messages were randomly presented on the digital display screen every hour. The messages that were used were based on previously effective studies and varied according to specified BCT; Table 1 outlined the verbatim description for each message. Data were collected over an approx. 14-week period between 12 August 2020 and 16 November 2020. Due to COVID restrictions changing throughout the study, two baseline data collection points were established to determine any alteration in usage seen might be due to this. The first was during stage 3 lockdown laws in Victoria, Australia and then again when restrictions eased to stage 1. In summary:

Step 1—Baseline from 12 August to 21 September 2020.
Step 2—Behavioral messages were rotated from 22 September to 6 October 2020.
Step 3—Baseline repeated from 7 to 13 October 2020.
Step 4—Behavioral messages were rotated from 14 October to 16 November 2020.

**Table 1:** List each message used and the associated BCTs.

Number	Screen message	BCT
Baseline	Hand sanitizer	Baseline
1	Be safe. Sanitize your hands.	Action planning
2	You must sanitize your hands.	Action planning
3	You should sanitize your hands.	Action planning
4	You can sanitize your hands.	Action planning
5	Please sanitize your hands.	Action planning
6	Most shoppers sanitize their hands.	Social comparison
7	Our shoppers sanitize their hands.	Social comparison
8	Usually our shoppers sanitize their hands.	Social comparison
9	Our shoppers usually sanitize their hands.	Social comparison
10	Washing hands with sanitizer avoids spread of COVID-19.	Information about health consequences
11	Washing hands with sanitizer can prevent the transmission of COVID-19.	Information about health consequences
12	Washing hands with sanitizer will prevent the transmission of COVID-19.	Information about health consequences
13	Clean hands prevent the spread of COVID-19.	Information about health consequences
14	Hand sanitizer: where have your hands been today?	Information about health consequences

Messages were randomly displayed from the message database.

### Statistical Analysis

Data was analyzed using SPSS Statistics version 25 (SPSS Inc., Chicago, IL), with a significance level set at *p* < 0.05 for all statistical analyses. Data were cleaned to encompass only the targeted dates. Records from the activity sensor, usage data and messages displayed were combined into one SPSS database by merging records based on hour and weekday. Duplicates were removed. The amount of activity and usage per hour was then totaled into a new variable titled “usage ratio,” which was calculated by computing the total dispenser usage per hour divided by the total activity sensor data per hour, multiplied by 100. The effect of both individual BCT message intervention and BCT groupings were assessed against the usage ratio. A one-way between groups analysis of variance (ANOVA) was applied to compare usage ratio against messages. Usage ratio was the dependent variable and messages being the independent variable. Hours of the day and day of the week were covariates.

## Results

Data was collected from 12 August 2020 to 16 November 2020. In total 63,657 customers entered the hardware store with a measured mean average for hand sanitization of 21.84% (CI = 21.18%–22.49%; Table 2). There was no significant change in baseline usage during timepoints reflecting changes to COVID-19-restrictions, however there was a significant difference in the rate of use for hour of the day *F*(10,361), 13.04, *p* < 0.001, and day of the week *F*(6,365), 4.30, *p* < 0.001, with the morning and weekends seeing the highest usage ratios.

**Table 2:** Descriptive statistics of each message type displayed on the hand sanitizer dispenser (mean and CI).

	*N*	Mean usage ratio	SD	Std. error	95% CI for mean	
					Lower bound	Upper bound
Baseline	460	21.24	9.85	0.459	20.34	22.14
1	24	21.89	11.96	2.44	16.84	26.94
2	37	23.66	10.62	1.75	20.15	27.23
3	44	22.91	10.02	1.51	19.86	25.95
4	29	19.73	10.23	1.90	15.84	23.62
5	25	23.12	9.94	1.99	19.02	27.22
6	32	25.13	9.16	1.62	21.83	28.43
7	33	23.53	12.22	2.13	19.20	27.86
8	31	20.97	10.16	1.83	17.24	24.70
9	30	22.66	10.04	1.83	18.91	26.41
10	37	20.81	10.13	1.67	17.43	24.19
11	29	24.78	10.62	1.97	20.74	28.82
12	34	23.93	9.64	1.66	20.56	27.29
13	29	21.47	10.06	1.87	17.65	25.23
14	30	19.08	9.51	1.74	15.53	22.63
Total	904	21.84	10.09	0.34	21.18	22.49

A one-way ANOVA was conducted to compare the effectiveness of messages on usage ratios. Weekday and hour of the day were entered as covariates due to their significance. Results showed that the usage ratio did not significantly change between individual messages and baseline [*F*(16,904) = 1.19, *p* = 0.279].

Messages were then grouped into their BCT. There was no significant difference in mean usage ratio either between BCT groups [*F*(3,906) = 1.33, *p* = 0.263].

**Figure 1:**
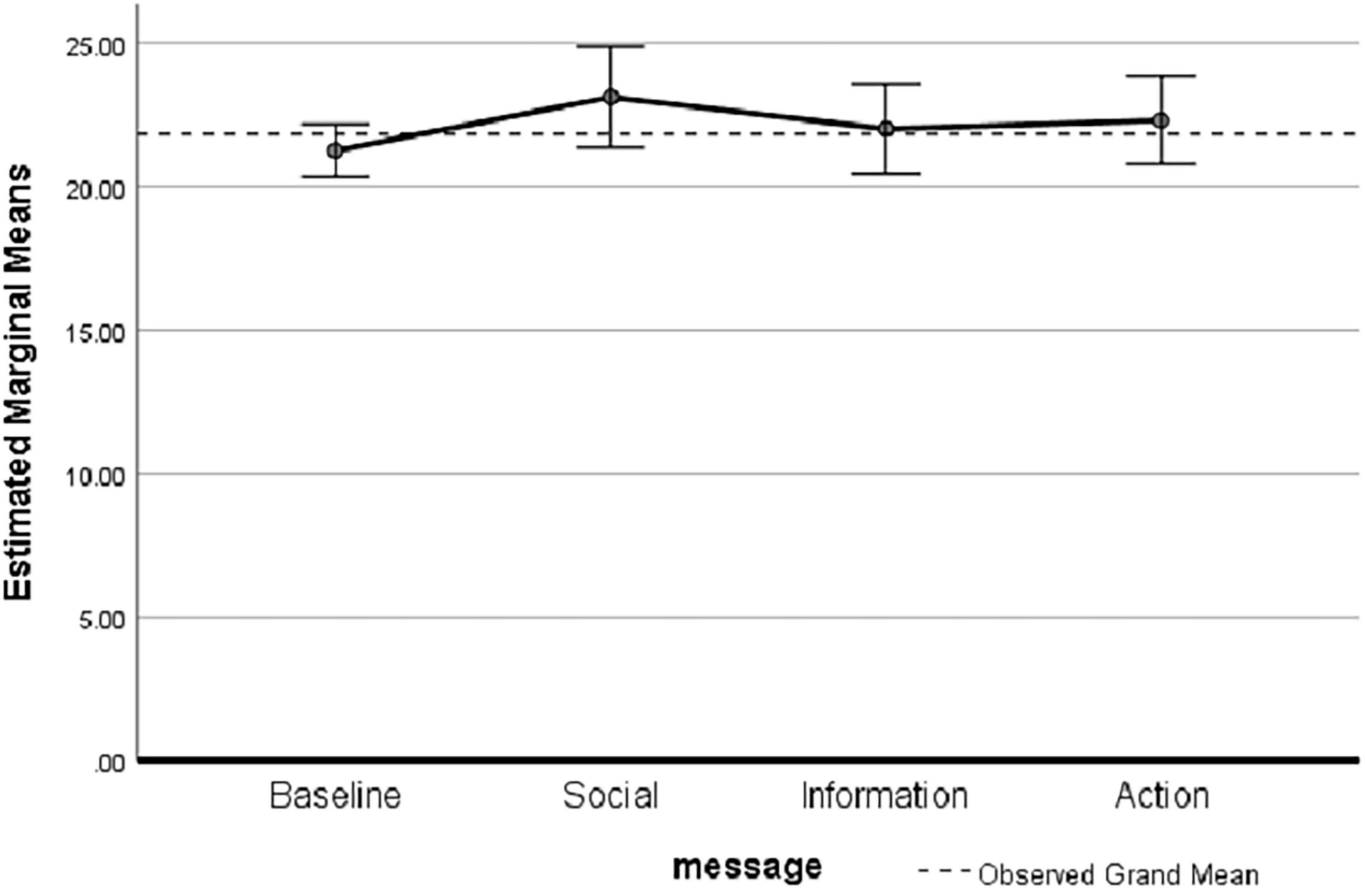
Estimated marginal means between usage ratio and behavioral change technique (BCT) message groups (*p* = 0.263; error bars = 95% CI, Covariates: hours = 12.04, weekday = 4.04).

*Post hoc* tests showed there was also no significant difference between messages (social comparison, *p* = 0.395; information, *p* = 1.000; action planning, *p* = 1.000; Figure 1).

## Discussion

This study found less than a quarter (~22%) of customers used hand sanitizer in a non-enforced environment, and the introduction of BCT health messages made no significant change to hand hygiene behavior in the COVID-19 pandemic context. The usage rates were similar to other studies conducted during a pandemic (2009 influenza; ~18%; Murray et al., 2009). These results suggest that the economic burden imposed on mandating hand sanitizer dispensers may be unnecessary given the lower-than-expected usage rates and the additional costs to smaller businesses; including installation, maintenance and refilling of stations, is the increased cost burden for small businesses worth the potential risk reduction of COVID-19 transmission?

Findings from this study indicate that different health messages, based on previously effective changes in hand hygiene behavior (Judah et al., 2009; Updegraff et al., 2011; Gaube et al., 2020), failed to have an impact on hand sanitizer usage in the COVID-19 pandemic context. Furthermore, there was no difference reported between baseline hand sanitizer usage and any BCT intervention. Consequently, if no association was found between the usage ratio and BCT messages, it could be concluded that a generic dispenser label (e.g., “hand sanitizer”) may sufficiently inform customers of their obligation to sanitize their hands. Results are not consistent with other research that have found that messages emphasizing social norms performed best (Judah et al., 2009) or that during the H1N1 pandemic, messages promoting a positive consequence to hand sanitization showed the greatest improvement in usage (Updegraff et al., 2011). The latter study however, measured only the amount of hand sanitization left in the dispensers to measure usage, not the actual usage ratio based on the amount of people passing by. Furthermore, a recently published article which randomly assigned one of five different behavioral messages (gain framing, social norm, guilt appeal, and exchange or a fifth control message) to participants found that simple, brief, and easily conveyable messages produced significantly higher intentions for participants to handwash (Matkovic et al., 2021); however, what people say they would do and what they actually do in a natural setting are potentially very different. Interestingly, in this current study, we did not observe any changes in usage over time, despite case numbers increasing and decreasing throughout the period, indicating that maybe the number of active and new cases might not have been a factor in the threat perception of individuals. Regional areas often have a closer network and sense of community than larger cities and as a result having only a few cases may be perceived as a higher risk since communities are much smaller.

Possible explanations for the null findings in this study could be that the signs were not big enough to engage people or to read the display messages, and thus did not impact on behavior. Also, the large, public health campaign already promoting hand sanitization behavior, may have negated any additional behavioral changes that could have been influenced by reading the different messages. There is also the possibility that based on the COM-B model of behavioral change theory, customers had the physical capability and were presented with the opportunity to hand sanitize but the motivation to was lacking (Michie et al., 2005). This might be due to some people using their own personal hand hygiene products, even though the directive was to hand sanitize on entering each store. In addition, some individuals might have had predetermined motivations or beliefs around hand sanitizing before entering, meaning that these people were not going to hand sanitize no matter what message was displayed. The positive is that hand sanitization was constant throughout the study and that the magnitude was higher than pre-pandemic studies (Judah et al., 2009; Updegraff et al., 2011).

The question that is raised from this study is what is considered optimal usage of hand sanitization? It is unclear what degree of hand sanitization is needed to be an effective public health intervention; therefore it is unknown whether 22% seen in this study was useful or not at reducing the risk and spread of COVID-19. Further research is needed to consider future policies and public health messages and to quantify critical hand sanitization usages rates.

It is probably unrealistic to expect 100% compliance, but quantifiable targets, to measure success of a hand hygiene intervention or public health policy, is needed. To our knowledge, there is very little data to inform the public and no guidance from the Centers for Disease Control and Prevention (CDC) on target usage to limit the spread of disease. Therefore, if usage is low, is it worth the economic burden placed on businesses to install, refill, maintain and have personnel continually monitor hand sanitizer dispensers and impose fines if businesses do not comply with these policies? Future research is needed to quantify the optimal level of hand sanitization (usage ratio) required during a pandemic for an effective public health intervention. Importantly, in subsequent months the CDC no longer regarded hand hygiene as the single most important public health strategy by de-emphasizing the need for environmental cleaning due to the low risk of transmission of COVID-19 from surfaces, highlighting the principal mode of transmission is *via* droplet transmission, direct contact or airborne transmission (Centers for Disease Control and Prevention, 2021). As such, hand sanitization would not be deemed as effective in stopping the spread of disease as would the use of face masks and social distancing. Thus, it might be unnecessary to continue to mandate Australian businesses, under current COVID-19 guidelines, to maintain hand sanitizing stations. Nonetheless, if increased hand sanitization in public settings is critical, but unviable to enforce; it raises questions for the contemplation of future policies, public health messages and the need to quantify usages rates. Further public health policies needed to consider the appropriateness and effectiveness of public health messages and rules in order to increase public safety without unnecessary burden on individuals and businesses.

### Strengths and Limitations

This was a novel; observational behavior change study; the research team acknowledge that there are some limitations. New technology was custom-built for this study and so the accuracy of the usage ratio was reliant on the reliability of the activity sensor and being able to distinguish between an individual and group of people at once moving together across its field of view. Both scenarios would have counted each of these events as only one person. As such, the number of customers recorded entering the store may have been more than recorded by the devices, thus, underestimating the number of people going through. Therefore, it may overestimate the ratio of hand sanitization, however as the entrance was not wide, most people would have gone through single file. Nevertheless, this is mitigated by the comparisons made between baseline and the various BCT messages. It is also possible that private or prior usage of hand sanitization before arriving at the store had an impact. Some patrons may have had a predetermined bias regarding behavior and response to public health messaging on entering a store, which cannot be controlled. In addition, due to these projects’ limited resources, recruitment was focused on one site, which reduces the generalizability, but results are comparable of past research that measured hand hygiene at one site and found similar percentages of usage. Overall, the results from the study are important as they highlight that, even during a pandemic with strong external public health messaging and heightened awareness of transmission of disease, hand sanitization rates did not change substantially. This indicates that different types of behaviorally constructed messaging is unlikely to change behavior, even during a pandemic.

### Conclusion

Frequent hand hygiene behavior has been promoted as necessary to limit transmission of COVID-19 and mandated for businesses during COVID-19-restrictions to provide hand sanitizer dispensers in Australia. However, what rate of community hand sanitizer usage is needed for it to be an effective public health intervention? This study found different messaging did not affect behavioral change, nor were hand sanitizer usage ratios higher, compared to other studies, even during a pandemic. Policy-wise, this raises questions on whether requirements imposed on businesses to provide hand sanitizer to patrons may be an ineffective and unnecessary economic burden. Future research should first focus on quantifying the optimal level of hand sanitization (usage ratio) required during a pandemic for an effective public health intervention, as there is insufficient data in the literature to inform the public.

## Data Availability Statement

The raw data supporting the conclusions of this article will be made available by the authors, without undue reservation.

## Ethics Statement

The studies involving human participants were reviewed and approved by the La Trobe University Human Research Ethics Committee (HEC20265). Written informed consent for participation was not required for this study in accordance with the national legislation and the institutional requirements.

## Author Contributions

LB, EC, HP, CF, SE, CC, and TS: conceptualization, methodology, supervision, and writing—review and editing. SE designed and built the new IoT technology. LB, EC, and TS: formal analysis and investigation. All authors contributed to the article and approved the submitted version.

## Conflict of Interest

The authors declare that the research was conducted in the absence of any commercial or financial relationships that could be construed as a potential conflict of interest.

## Publisher’s Note

All claims expressed in this article are solely those of the authors and do not necessarily represent those of their affiliated organizations, or those of the publisher, the editors and the reviewers. Any product that may be evaluated in this article, or claim that may be made by its manufacturer, is not guaranteed or endorsed by the publisher.
